# Data Processing Thresholds for Abundance and Sparsity and Missed Biological Insights in an Untargeted Chemical Analysis of Blood Specimens for Exposomics

**DOI:** 10.3389/fpubh.2021.653599

**Published:** 2021-06-10

**Authors:** Dinesh Kumar Barupal, Sadjad Fakouri Baygi, Robert O. Wright, Manish Arora

**Affiliations:** Department of Environmental Medicine and Public Health, Icahn School of Medicine at Mount Sinai, New York, NY, United States

**Keywords:** LC/MS, exposome, metabolomics, untargeted chemical analysis, birth weight

## Abstract

**Background:** An untargeted chemical analysis of bio-fluids provides semi-quantitative data for thousands of chemicals for expanding our understanding about relationships among metabolic pathways, diseases, phenotypes and exposures. During the processing of mass spectral and chromatography data, various signal thresholds are used to control the number of peaks in the final data matrix that is used for statistical analyses. However, commonly used stringent thresholds generate constrained data matrices which may under-represent the detected chemical space, leading to missed biological insights in the exposome research.

**Methods:** We have re-analyzed a liquid chromatography high resolution mass spectrometry data set for a publicly available epidemiology study (*n* = 499) of human cord blood samples using the MS-DIAL software with minimally possible thresholds during the data processing steps. Peak list for individual files and the data matrix after alignment and gap-filling steps were summarized for different peak height and detection frequency thresholds. Correlations between birth weight and LC/MS peaks in the newly generated data matrix were computed using the spearman correlation coefficient.

**Results:** MS-DIAL software detected on average 23,156 peaks for individual LC/MS file and 63,393 peaks in the aligned peak table. A combination of peak height and detection frequency thresholds that was used in the original publication at the individual file and the peak alignment levels can reject 90% peaks from the untargeted chemical analysis dataset that was generated by MS-DIAL. Correlation analysis for birth weight data suggested that up to 80% of the significantly associated peaks were rejected by the data processing thresholds that were used in the original publication. The re-analysis with minimum possible thresholds recovered metabolic insights about C19 steroids and hydroxy-acyl-carnitines and their relationships with birth weight.

**Conclusions:** Data processing thresholds for peak height and detection frequencies at individual data file and at the alignment level should be used at minimal possible level or completely avoided for mining untargeted chemical analysis data in the exposome research for discovering new biomarkers and mechanisms.

## Introduction

Small molecules can function as metabolic substrates and products, signaling molecules, energy equivalents, building blocks, and toxic exposures in the human body (1). Beneficial and harmful changes in the levels of these molecules can occur from the early life to the elderly stage because of genetic and environmental factors (2–5). Inside the human body, a small molecule can be absorbed, circulated, metabolized, degraded, transformed and excreted by organ systems (6, 7). A comprehensive understanding of these changes and how the body's biological pathways react to them can contribute to advancing prevention strategies for human diseases (8, 9). But, contrary to the vast knowledge available on functional roles of genes or proteins, our understanding about the roles of most small molecules in human health remains rudimentary (10, 11). Fortunately, use of sensitive and high-resolution analytical techniques such as a gas or liquid chromatography mass spectrometry (GC/MS or LC/MS) to detect small molecules in bio-fluids in a comprehensive manner can expand our knowledge about relationships between small molecules and human health (5, 11, 12).

Advanced and cost-effective nucleic acid sequencing methods can identify rare DNA variants, RNAs, and protein variants to improve our understanding about the development and spread of diseases (13–15). Similarly, untargeted chemical analyses using a high-resolution LC/MS instrument can detect thousands of small molecules in bio-fluid samples and can generate high quality and rich semi-quantitative data to identify and discover new metabolic hypotheses, biomarkers, and risk factors for diseases and biological phenotypes (16–20). In contrast to a targeted assay (21), an untargeted assay aims to detect in an unbiased manner as many possible chemicals present in a bio-fluid sample using a GC/LC-HRMS instrument (18) and such untargeted assays are key methods in exposome research to identify and discover new risk factors and biomarkers for chronic diseases (21–23).

However, untargeted chemical analyses have mandatory laboratory and computational biases (24) which decide the composition of the data matrix that will be used for association analyses. Laboratory biases include the extraction solvent, sample re-suspension method, type of GC or LC column, chromatography gradient, ionization mode and parameters and mass spectrometry detector (25–27). Computational biases include various data processing thresholds that were used during peak detection, peak annotation, alignment and gap filling steps. For the exposome research, it is important that all possible small molecules in a specimen are reliably captured in the data matrix to characterize the dark matter or missing biology of the etiology of human diseases (11). While it is difficult to fix laboratory biases without re-analyzing the bio-fluid samples, computational biases can be readily addressed by reanalyzing collected raw LC/GC-HRMS data with minimum thresholds during data processing steps. Minimizing the effect of these biases can increase the number of chemical signals that can be tested for having an association with a phenotype as highlighted in computational method optimization studies (28–34).

**Figure 1:**
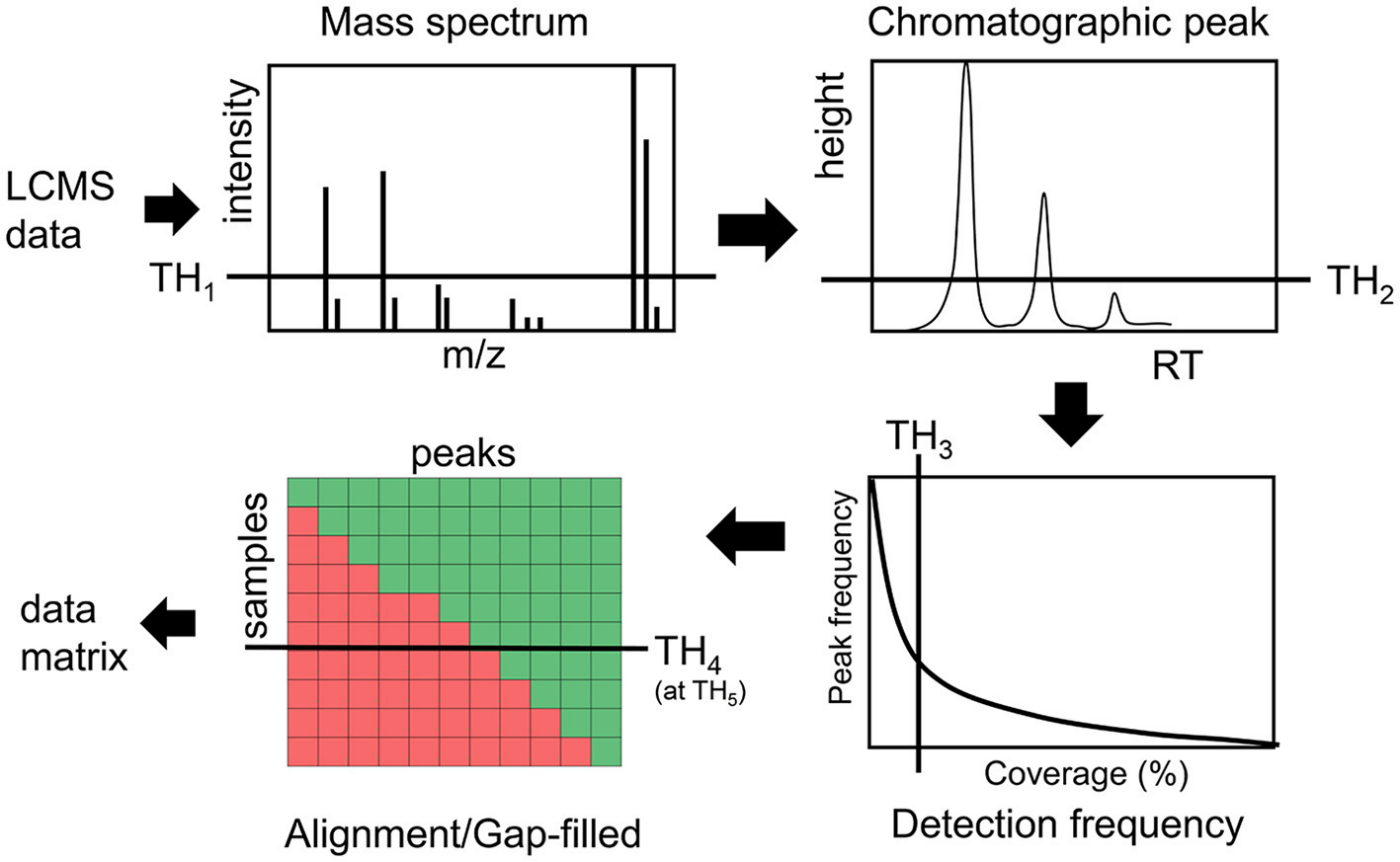
Overview of different thresholds in processing LCMS data in untargeted metabolomics assays.

Intensity and detection frequency thresholds during the peak detection and peak alignment steps have the strongest effect (35–37) on the data matrix composition (Figure 1). During the peak detection step, an intensity threshold TH_1_ rejects mass to charge (m/z) values in each scan, then a peak height threshold TH_2_ rejects chromatographic peaks in individual LC/MS file. In the next step, peaks are aligned and gap-filled across multiple files if they pass the peak height threshold TH_2_ in a minimum number of samples defined by a threshold TH_3_. During the gap-filling process, relaxed criteria are used to recover peaks from raw data. Then finally peaks are rejected if they were found to be missed at a threshold TH_4_ on an intensity threshold TH_5_ (Figure 1). While these thresholds are used in almost every untargeted metabolomics study, there are no guidelines and standards to justify them, leading to a large variation in data matrix sizes from one study to another study for the same type of biospecimen (38).

**Figure 2:**
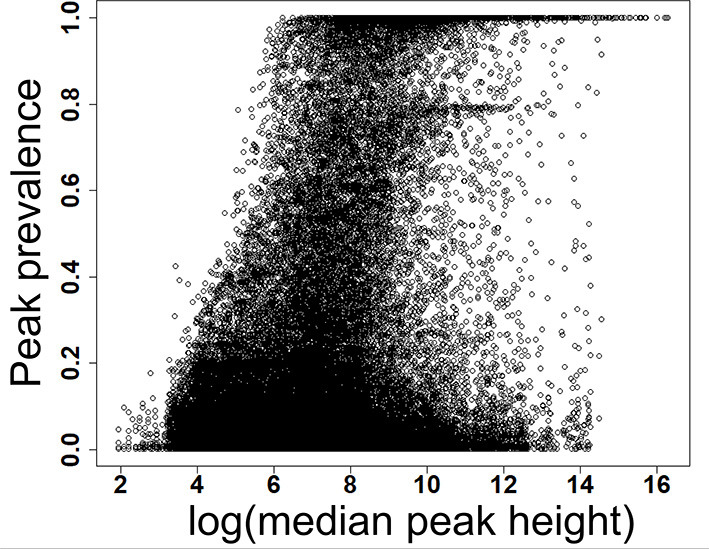
Prevalence of low and high abundant peaks across 499 data files. X-axis shows the scaled detection prevalence, where 1.0 means a peak was detected in all the samples. Y-axis shows the median peak height across all files for a peak. MS-DIAL parameters are available in the Supplementary Table 1.

In this report, we have re-analyzed a publicly available untargeted LC/MS data for 499 cord blood samples (35, 39) using the MS-DIAL software (40) with minimum possible thresholds during data processing, and highlighted how different levels of these thresholds (Figure 2) alter the data matrix composition. We also highlight that a practice of stringent thresholds can miss metabolic hypotheses about a phenotype of interest such as newborn birth weight. Our results underscore the need to avoid or to use minimally possible values for these thresholds when processing untargeted LC/MS datasets for exposomics research.

## Methods

### Dataset

We have utilized a publicly available large untargeted metabolomics dataset of cord blood (35, 39). The dataset was collected using an Agilent 1290 Infinity Liquid chromatography system connected to an Agilent quadruple time of flight (qToF) 6550 mass spectrometry instrument in the ESI positive mode. The details of the sample preparation, LC and MS conditions are provided in the original article (39) and at the MetaboLights database (accession number MTBLS1684). In brief, metabolites from a cord blood sample were extracted using acetonitrile and separated by ACQUITY UPLC HSS T3 (2.1 × 100 mm, 1.8 μm; Waters) reversed phase column using methanol as solvent B. A pooled QC sample was injected after every 12 injections. The original analysis was conducted using a workflow of the Agilent Qualitative Analysis B.06.00, DA Reprocessor, and Mass Profiler Professional 12.1 software. Briefly, in the original analysis, compounds were detected as proton adducts (M+H)+ using the recursive molecular feature extraction algorithm with a mass intensity threshold of 1,500, a chromatographic peak height threshold of 10,000 and a mass tolerance threshold of 0.0025 for isotope pattern matching. Features that were detected in at least 2% of all the samples were aligned using a RT tolerance of 0.075 and a mass tolerance of 15 ppm + 2 mDA. Aligned features were used as target list for recursive feature extraction using the find by formula algorithm with a mass tolerance of 10 ppm and 0.04 min. Finally, features that would not present in at least 60% of samples were removed.

We have downloaded the raw data files in the Agilent.d format from the EBI MetaboLights database. Data files were converted to the mzML format using the ProteoWizard MSConvert software version 3.0.20183 with binary encoding precision of 64-bit, zlib compression, write index and TPP compatibility enabled. Birth weight data were available for all the samples. Original reported untargeted data matrix was also downloaded from the MetaboLights repository.

### LCMS Data Processing

We have used the MS-DIAL software to perform peak-detection, alignment and gap filling for the mzML files. MS-DIAL parameters are provided in the Supplementary Table 1. Key MS-DIAL parameters were—MS1 tolerance: 0.01 Da, Retention time tolerance: 0.05 min, MS1 tolerance: 0.015 Da, *N*% detected in at least one group: 0, Smoothing method: LinearWeightedMovingAverage, Smoothing level: 3, Minimum Peak Height: 0 and Minimum peak width: 5. Individual peak list for all 499 files and alignment results for peak height were exported tab delimited text files from the MS-DIAL software. Individual peaks lists and the aligned peak table are provided in the Figshare repository at https://doi.org/10.6084/m9.figshare.13564823.v1 and https://doi.org/10.6084/m9.figshare.13564838.v1. MS-DIAL also computed peak height, peak area, signal to noise ratio (S/N) for each detected peak. The % fill value column indicated that proportion of files in which a peak was detected with a high confidence, where 1.0 means the peak was present in all the samples before the gap-filling step. De-isotoping was performed by MS-DIAL with default settings. We did not perform any adduct grouping on the aligned tables, which may have penalized the *p*-value adjustment for multiple hypotheses testing, however the rank order of top fifty significant peaks and our conclusions are unaffected by it.

### Statistics and Graphics

The MS-DIAL generated data matrix was imported in the R software version 4.0.3. For each LC/MS peak, min, max, average and standard deviation were computed after removing the zero values. For the peak list of each file, peak heights were divided into 20 bins and upper boundaries for those bins were obtained. Signal to noise (S/N) ratio as computed by MS-DIAL were extracted from each peak-list and divided into 20 bins similar to the steps taken for peak height summarization. For individual files and the final data-matrix, peak height thresholds of 1,000, 2,000, 5,000, 10,000, and 20,000 were used and corresponding coverage of peaks across all files were obtained. Correlation between the peak height and birth weight was computed using a spearman coefficient for all the peaks. *P*-values obtained for the spearman analysis were adjusted for multiple hypothesis testing using the Benjamini & Hochberg false discovery rate approach in R. Graphics were prepared by R and Microsoft Excel.

## Results

### Effect of Peak Height Thresholds on the Peak List at Individual File Level

#### Descriptive Summary

We have re-analyzed 499 LC/MS data files available from a publicly available cord blood metabolomics study [EBI MetaboLights accession number MTBLS1684 (39)] using MS-DIAL (40), an universal and free software for processing GC and LC/MS data for metabolomics and lipidomics analyses. This study was chosen as a case study because of it's large sample size, but it has a negligible drift in retention time and mass spectrometry signals (Supplementary Figures 1–5). The published data matrix for this study was generated using Agilent Mass Hunter Qual and Mass Profiler Professional software using stringent thresholds for peak detection, alignment, and detection frequency (39) during the data processing workflow (see section Methods). For this case study, MS-DIAL detected an average of 23,156 (±1,385) peaks across all files when the lowest possible thresholds for data processing were used (see section Methods). We have chosen peak height as an abundance indicator for a peak because it is minimally affected by baseline fluctuations in chromatography. For the case study, the highest peak height was 12,392,001, giving the lowest reliable point in the dynamic range as 1,239 peak height because of the four order of dynamic range for Agilent QTOF instruments. Across all the 499 files, 80% of peaks had an average peak height <8,428 (±11.2%), indicating that a vast majority of detected peaks in those files were low abundant (Table 1 and Figure 2) and within the dynamic range of the QTOF instrument.

**Table 1:** Distribution of peak height values across 499 files.

**Bin (%)**	**Average peak height boundary**	**Relative standard deviation (%)**
95–100	12,392,001	8.2
90–95	50,518	9.5
85–90	21,326	10.2
80–85	12,521	10.6
75–80	8,428	11.2
70–75	6,069	11.7
65–70	4,588	12.0
60–65	3,576	12.3
55–60	2,837	13.0
50–55	2,274	13.5
45–50	1,829	14.1
40–45	1,467	14.9
35–40	1,158	16.1
30–35	890	17.6
25–30	657	18.9
20–25	466	19.1
15–20	322	17.2
10–15	222	13.5
5–10	152	9.7
0–5	99	6.3
0–0	0	0.0

*It provides an overview of the how the use of different peak height thresholds can decide the composition of peak list at individual LC/MS data file level*.

Since, we have used minimally possible thresholds in the data processing to retain low abundant peaks, the data quality of the detected peaks needed to be evaluated. But it is not feasible to manually inspect each peak in each file by plotting an extracted ion chromatogram, so signal to noise ratio (S/N) parameter was used as a gross indicator of the peak quality and tabulated for all detected peaks. We observed that across all the 499 files, at least 50% peaks in each file had a S/N of at least 5, indicating that detected low abundant peaks had a reasonably good quality (Table 2).

**Table 2:** Distribution of S/N ratio across 499 files.

**Bin (%)**	**Average S/N boundary**	**Relative standard deviation (%)**
95–100	31,957.6	14.7
90–95	95.0	8.7
85–90	43.9	8.8
80–85	28.0	9.3
75–80	20.3	9.3
70–75	15.7	9.5
65–70	12.7	9.4
60–65	10.6	9.4
55–60	8.9	9.3
50–55	7.6	9.2
45–50	6.5	9.2
40–45	5.5	9.1
35–40	4.7	9.0
30–35	4.0	9.0
25–30	3.4	9.0
20–25	2.8	10.1
15–20	2.2	10.8
10–15	1.7	9.5
5–10	1.2	7.3
0–5	0.9	5.2
0–0	0.0	0

Next, we assessed how different peak height thresholds can impact the peak list composition at individual file level by counting the rejected peaks by a threshold and a S/N ratio of at least 2. At the peak height threshold 10,000 which was used by the MassHunter software in the original publication, 64% peaks (Table 3) on average across all files were rejected, suggesting that a majority of low abundant peaks were excluded from the statistical analysis in the original publication.

**Table 3:** Peak rejections by different peak height thresholds on the individual file level.

**Peak height cutoff**	**Average % rejection of peaks with S/N ratio of ≥2**	**Standard deviation**
100	0.15	0.06
200	1.90	0.30
500	10.00	1.16
1,000	20.34	1.60
2,500	39.72	1.54
5,000	53.77	1.09
10,000	64.15	1.09

### Effect of Peak Height and Detection Frequency Thresholds on the Aligned Peak Table Composition

For the case study, MS-DIAL generated an aligned and gap filled data matrix which had a total of 63,393 peaks, representing the detected chemical space for the study in a comprehensive manner. We asked how many of those peaks will be rejected from this data matrix if they were not found in at least one LC/MS data file at a peak height threshold (Table 4). At a peak height threshold of 10,000 peak height which was used in the original publication, up to 70% peaks were rejected.

**Table 4:** Effect of peak height thresholds on the peak rejection at the peak alignment level.

**Peak height threshold at the alignment level**	**Rejected peaks (total)**	**Percentage (%)**
20,000	51,924	81.9
10,000	44,331	69.9
5,000	33,444	52.8
4,500	31,404	49.5
4,000	28,998	45.7
3,500	26,194	41.3
3,000	22,781	35.9
2,500	18,493	29.2
2,000	13,079	20.6
1,500	6,626	10.5
1,000	4,273	6.7
500	2,910	4.6
200	878	1.4
100	71	0.1

*A peak was rejected if it failed to pass a given peak height threshold in all files*.

**Figure 3:**
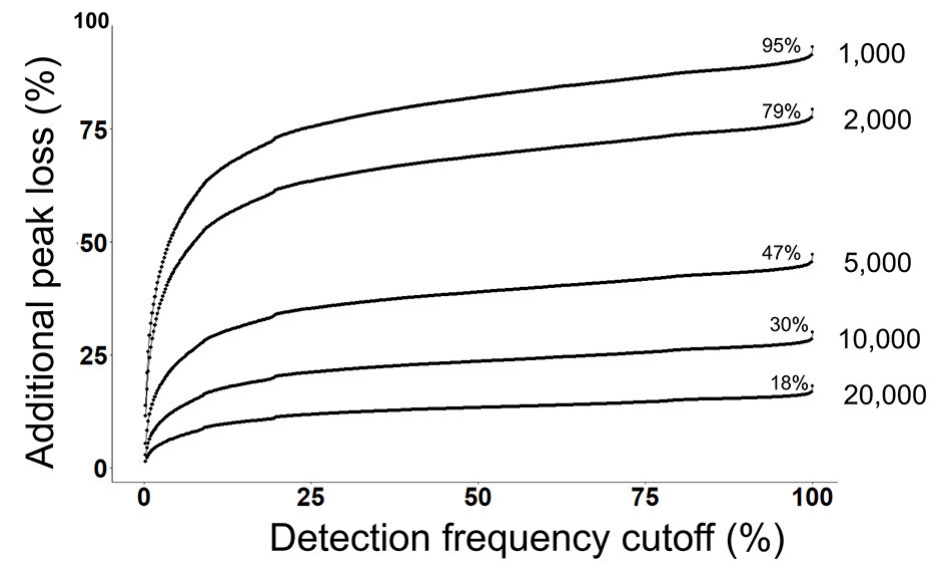
Detection frequency cutoffs and peak rejection in LC/MS data processing. For each peak height cutoff (1,000, 2,000, 5,000, 10,000, and 20,000) (TH2), a count of total retained peaks which were found in at least one sample was obtained as shown by the % values on each curve. Y-axis shows the loss of peaks as a function of detection frequency cutoffs. For example, at a detection frequency cutoff of 50% and peak height cutoff of 10,000, additional 20% peaks will be lost, retaining only 10% peaks.

Next, we evaluated that how many peaks will be rejected by a combination of both a detection frequency threshold (TH3) and a peak height threshold (TH2) across all 499 files at the alignment level (Figure 3). We use the MS-DIAL provided % fill property as an indicator of the positive detection of a peak in the sample. On a 2% detection frequency threshold (TH3) along with a 10,000 peak height threshold, as it was used in the original publication, additional 5% peaks were rejected, retaining only 25% peaks at the 10,000 peak height threshold in the aligned peak table.

Commonly used statistical methods and data normalization strategies require that input data matrices are not sparse, therefore missing values are replaced with an imputed value (41). The input for missing value imputation is generated by constraining the aligned peak table by a global peak detection frequency threshold (TH4). Often, peaks that were missing in >50% samples in the aligned table are rejected before the missing value imputation step. For the case study, in the original publication, peaks were rejected if they were not found in at least 60% of samples. At this threshold, 20% additional peaks were rejected, leaving only 10% peaks that have passed the 10,000 peak height cutoffs (Figure 3).

Overall, a combination of peak height (TH2) and detection frequency thresholds (TH3 and TH4) that were used in the original publication has rejected 90% of peaks which were defined by MS-DIAL for the case study. These results suggest that the informatics strategy that was used in the publication may have missed many metabolic hypotheses in relation to birth weight.

### Data Processing Thresholds and Missed Biological Insights

**Figure 4:**
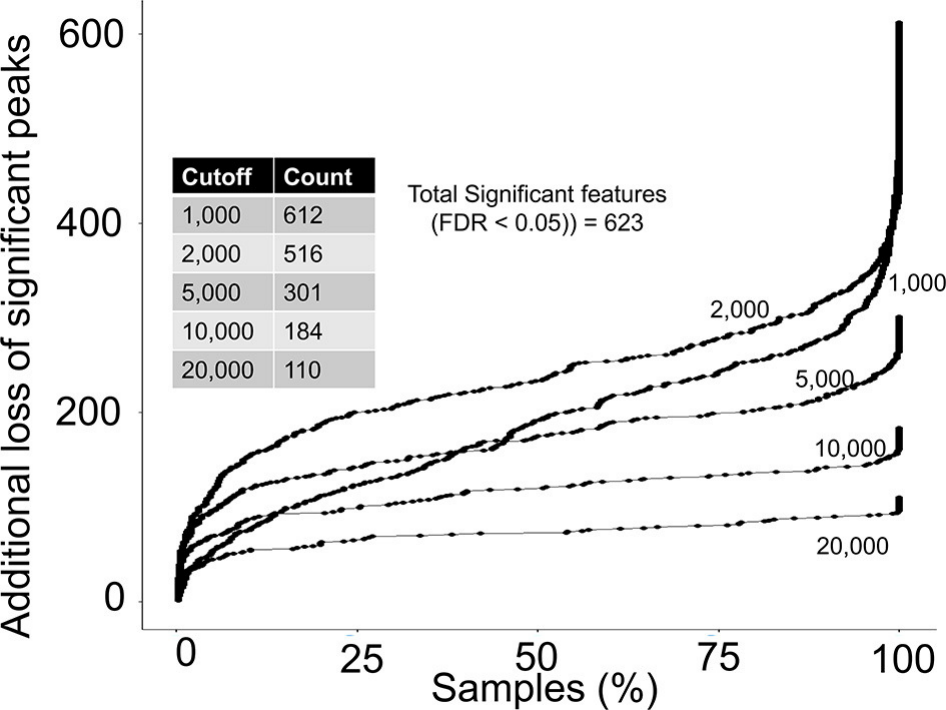
Rejection of biological relevant peaks by peak height and detection frequency thresholds. For each peak height threshold (1,000, 2,000, 5,000, 10,000, and 20,000), a count of total peaks that were associated with birth weight are shown in the inside table. Y-axis shows the additional loss of peaks as a function of detection frequency cutoffs. For example, at a detection frequency cutoff of 50% and peak height cutoff of 10,000, additional 100 significant peaks will be lost, yielding retainment of only 84 peaks.

Next, we asked if any of the rejected peaks were associated with birth weight by computing spearman correlation co-efficient between peak height of each peak and birth weight data. Since, none of the co-variate data for the study participants were publicly available at the MetaboLights entry for the study, the correlation analysis can be considered as a crude or un-adjusted linear regression model. The correlation analysis suggested that 623 peaks in the complete aligned peak table were significantly associated with birth weight after adjusting for multiple hypothesis testing by a false discovery rate (FDR) cutoff of 0.05. On a peak height threshold of 10,000, 80% of those significant peaks were rejected (Figure 4) which was consistent with the overall peak rejection rate (Figure 3). The results highlighted that a combination of peak height and detection frequency thresholds has rejected peaks that indicated significant associations between metabolic pathways and birth weight.

A complete list of peaks that were significantly associated with birth weight is provided in Supplementary Table 2. Top 50 significant peaks are shown in the Table 5 to demonstrate few examples of missed metabolic insights because of stringent data processing thresholds. First three peaks share the same retention time, suggesting they may have fragmented from a single compound in the ESI ion-source. The peak of m/z 412.3035 and RT 5.75 min is a putative hydroxy acyl-carnitine, indicating that acyl coenzyme A-dehydrogenases may play a role in birth weight. The peak of m/z 289.2162 and RT 4.83 min. is a putative C19 steroid hormone, probably testosterone which have been previously linked with birth weight (42–44). Both of these peaks passed the 60% coverage cutoff (TH4) but failed to pass the 10,000 peak height in 2% sample cutoff (TH3) and therefore were missed in the original publication's statistical analysis (Table 5). Querying the m/z values for top 50 peaks against the Metabolomics Workbench RefMet database (45) suggested that a majority of them belong to lipid, acyl-carnitine and aromatic chemical classes. We cannot confidently annotate these peaks with chemical structures without analyzing the authentic standards using the same analytical conditions, these suggestive chemical annotations can be useful to derive new metabolic hypotheses for the birth weight and to conduct follow up studies.

**Table 5:** Top 50 peaks that were significantly associated with birth weight using a spearman correlation coefficient corrected for multiple hypothesis testing using the FDR approach.

**Retention time**	**m/z**	***p*-value (birth weight)**	**Maximum peak height**	**Passed 60% detection threshold**	**Passed the 10,000 peak height in in at least 2% samples threshold**
3.664	549.1632	3.12E-12	3,703	No	No
3.663	487.1944	2.25E-11	5,983	No	No
3.665	518.1235	9.11E-11	1,624	No	No
5.566	416.3373	1.52E-10	4,241	No	No
5.751	412.3035	1.62E-10	11,160	Yes	No
8.868	402.7667	3.32E-10	17,350	Yes	Yes
8.866	788.5606	3.61E-10	14,002	Yes	Yes
3.655	482.2388	3.88E-10	34,862	Yes	Yes
3.662	429.1907	8.25E-10	3,790	No	No
4.834	289.2162	8.61E-10	11,937	Yes	No
9.05	766.5737	1.25E-09	5,761	Yes	No
4.704	351.5335	2.27E-09	1,564	No	No
8.87	394.7769	2.81E-09	3,892	No	No
4.703	326.5377	4.22E-09	1,922	No	No
5.702	456.3321	4.23E-09	2,804	No	No
4.225	390.2097	4.75E-09	8,354	No	No
8.399	407.766	5.07E-09	2,780	No	No
8.402	776.5789	5.25E-09	3,936	No	No
5.796	367.157	5.64E-09	4,124	No	No
0.613	144.1254	6.27E-09	11,772	Yes	No
0.813	287.2451	8.31E-09	2,335	No	No
0.599	390.3455	8.39E-09	6,976	No	No
8.961	386.7638	8.64E-09	21,069	Yes	Yes
8.863	766.5765	9.28E-09	60,338	Yes	Yes
9.078	385.3477	1.10E-08	50,276	Yes	Yes
4.704	567.123	1.32E-08	8,785	No	No
4.704	582.0884	1.43E-08	10,873	Yes	No
8.961	756.5529	1.65E-08	12,703	Yes	No
4.705	342.5484	1.81E-08	3,836	No	No
5.648	463.2338	1.84E-08	15,180	Yes	Yes
4.846	475.1308	2.74E-08	2,328	No	No
5.307	464.6273	2.91E-08	1,382	No	No
3.828	274.0564	3.24E-08	2,644	No	No
8.962	734.571	3.95E-08	44,901	Yes	Yes
4.71	328.0402	4.45E-08	2,742	No	No
0.606	287.2457	4.93E-08	116,177	Yes	Yes
4.703	550.0624	5.80E-08	3,398	No	No
9.466	416.7825	5.81E-08	3,475	No	No
4.704	482.1513	5.84E-08	15,513	Yes	No
4.705	310.5219	6.33E-08	2,155	No	No
8.958	453.8122	6.82E-08	1,363	No	No
6.279	492.3678	6.96E-08	1,687	No	No
4.7	587.0983	7.11E-08	2,010	No	No
4.838	271.2058	7.36E-08	9,064	Yes	No
3.831	358.0548	7.46E-08	2,924	No	No
4.703	487.1076	7.86E-08	14,291	Yes	No
5.311	503.1375	8.51E-08	3,875	Yes	No
9.076	407.3296	1.03E-07	29,957	Yes	Yes
4.704	535.0978	1.06E-07	4,169	No	No

*Sixty percentage threshold indicates that the peak was found in at least 300 samples and the last column indicate that the peak must have a 10,000 peak height in at least 10 samples. These two thresholds were used in the original publication (39)*.

## Discussion

Untargeted chemical analysis of blood specimens has a great potential in the exposome research to expand our understanding about the hazardous effect of chemical exposures, to identify risk factors for disease and to identify biomarkers for physiological disturbances. Our analysis highlights how various data processing thresholds affect the comprehensiveness of the data matrix in untargeted chemical analysis of human blood samples in a large epidemiology scale study. Our key conclusion is that to maximize the impact of untargeted chemical analyses in the exposome and in broader metabolomics research, different thresholds that are used in the data processing workflows (Figure 1) should be avoided or used at a minimally possible level. We have shown that how a combination of these thresholds has rejected peaks that could provide new insights into the biological relationships among C19 hormone, hydroxylated acyl-carnitines and birth weight. Poorly explored and under-represented untargeted chemical analysis datasets are missing opportunities to expand our understanding about hazardous effect of chemical on human health.

Low abundant and non-ubiquitous signals precisely reflect the exposome phenotype of an individual (46). Small molecules at a low concentration tends to carry more risk for diseases (47, 48), need regulatory guidelines and biomonitoring (49, 50), are biologically more active and several are toxic. Whereas, high abundant and ubiquitous small molecules in blood are originating from normal endogenous metabolic pathways, carry less risk and are less toxic (11, 51). Data processing thresholds can inappropriately reject peaks for these highly relevant compounds in the former category for exposome research. A previous study has highlighted the negative effect of these thresholds on comprehensiveness (52) of untargeted data matrices, but it has not yet been demonstrated for a large-scale study with ~500 samples.

A highly sparse data matrix with the peak distribution skewed toward low abundant peaks realistically represent the expected blood exposome in an epidemiology study. There are several reasons to support it. (1) limit of detection for the method and analytical instruments so a peak will be detected only in samples in which it was sufficiently abundant (2) many endogenous compounds are found at the low abundant level because of the evolutionary advantages of enzyme promiscuity, providing structurally diverse low abundant compounds (53–55) (3) intermediate substrates in a metabolic pathways have a short life-time and less accumulation because of high affinity of enzymes in non-rate limited steps (4) many compounds are not ubiquitous and show a tissue and organ specificity so they can only be detected in blood samples from individuals with organ specific damages (56). (5) Exogenous compounds correlate with diet, exposures and other lifestyle factors which are known to vary greatly among general population (5). Therefore, for a blood or urine analysis, it is expected that many small molecules will be found in few samples and often on the low abundance levels.

A majority of exposome chemicals are at low abundance and tend to be less ubiquitous (46). It is a common situation that exposures above a threshold level can be present in blood specimens for only a few individuals (57, 58) and majority of the population will not be exposed to certain chemical unless we are studying a focused population such as metal-industry workers and firefighters. It is known that commonly used LC/MS instruments can have a four to five orders of dynamic range (52) and limit of detection (LoD) for an untargeted assay can be 0.02 ng/l for environmental pollutants (59). Any chemical compound present in a human biospecimen can be considered an exposure for an exposome wide-association study and a 11-order magnitude of concentration range is expected for compounds in the human blood (11). An untargeted LC/MS assays using an instrument having 4–5 order dynamic range and an optimized sample preparation can cover a significant portion of the exposome and metabolome, but a use of higher intensity thresholds and detection frequency threshold can miss many relevant compounds that were actually presented in the raw data. Merely, the presence of a hazardous chemical in an untargeted chemical analysis data of a blood specimen can be alarming since it shows that the level is higher than the expected LoD of that assay which can be higher than the safe level of an exposure.

We acknowledge that the annotation of low abundant peaks is a major challenge in the field of metabolomics and exposomics. It needs a holistic approach to get proper experimental MS/MS spectra and to prioritize their candidate structures for a confirmation by authentic standards (60–62). Furthermore, several detected peaks can correspond to in-source fragments, artifact and solvent contaminants, and removal to those peaks require a data filtering strategy such as blank subtraction or filtering by inconsistent intensities in a quality control sample. However, a peak that is strongly associated with a phenotype of interest will unlikely to be corresponding to a non-biological chemical compound. The critical limitation of a quality control (QC) sample based filtering and normalization approach is that it does not work for sparse matrices and excludes compounds that are low abundant and have a lower detection frequency. Our analysis underscores the need for development of data normalization strategies for sparse matrices which may or may not have a compound present in a QC sample.

Because quality control or blank samples were not submitted in the EBI Metabolights entry for the study, we are limited in terms of filtering data by additional methods. But a negligible drift in the ESI signal in total ion chromatograms and extracted ion chromatograms across 499 files was observed (Supplementary Figures 1, 2), suggesting that a QC based correction was not needed for this study and importantly a peak that is significantly associated with a phenotype will have a minimum likelihood of being falsely detected. MS-DIAL may have included some spurious peaks in the data matrix because of lower TH1–TH4 thresholds. However, the gain of using minimum levels of these thresholds out-weighs the risks of having few extra spurious peaks, as it finds more potential biomarkers and the ones that are strongly correlated with a phenotype as we have shown in the manuscript. Since, the primary application of untargeted LC-MS assays is to generate new hypotheses, a false-negative rate is more concerning.

These data processing thresholds were originally developed for processing untargeted chemical analysis data to study central metabolic pathways or universal pathway such as Krebs cycle intermediates (63) or lipid metabolites (64–66), but exposome research warrants that we detect and report all the chemicals present in the untargeted data at any possible level and at even in one sample. There has been development in the direction to build automated workflows that reject peaks using machine learning models (67) and rule based approaches (68), and those tools can be used to filter out low abundant peaks with a poor quality.

## Conclusion

Low abundant and less ubiquitous compounds in untargeted chemical analysis is an under-mined repertoire of missing biology and metabolic hypotheses in the metabolomics and exposome research. However, use of different thresholds during peak detection, alignment and statistics steps to reject LC/MS peaks constraints the untargeted analysis to only ubiquitous and core metabolic pathways. Therefore, for the exposome research, untargeted chemical analysis data matrices should be generated using minimally or no thresholds during data processing which can be achieved using MS-DIAL, a freely available software for processing epidemiology scale untargeted LC/MS datasets. A re-analysis of the case study data suggests a role of C19 steroids and acetyl dehydrogenase enzymes in regulating the birth weight, a biological insight that was missed in the original publication.

## Data Availability Statement

The case study are available at the Metabolights database with accession number MTBLS1684. Peak lists generated by MS-DIAL are available in the Figshare repository https://doi.org/10.6084/m9.figshare.13564823.v1. Additional data are provided in the Supplementary Material of the paper.

## Author Contributions

DB and SB planned and conducted the analysis. All authors contributed to the manuscript writing.

## Conflict of Interest

The authors declare that the research was conducted in the absence of any commercial or financial relationships that could be construed as a potential conflict of interest.

## Supplementary Material

Supplementary Figure 1 A negligible drift in the ESI signal across 499 files. Calibration compound (m/z = 922.0098) was injected on a constant flow rate by a secondary HPLC pump directly into the ESI ion-source. In case of a significant signal drift, a gray to black gradient in this plot would appear according to the injection order. Absence of such gradient shows a negligible signal drift in the study.

Supplementary Figure 2 A negligible drift in the ESI signal across 499 files as demonstrated by overlaying total ion chromatograms. In case of a significant signal drift, a gray to black gradient in this plot would appear according to the injection order. Absence of such gradient shows a negligible signal drift in the study.

Supplementary Figure 3 Demonstration of a negligible drift in retention time for the peak of PC 32:0 (m/z 734.57).

Supplementary Figure 4 Demonstration of a negligible drift in retention time for the peak of tryptophan (m/z 205.0976).

Supplementary Figure 5 Demonstration of a negligible drift in retention time for the peak of AC 16:1 (m/z 398.3264).

Supplementary Table 1 MS-DIAL parameters that were used for processing the LCMS data files.

Supplementary Table 2 Correlation between birth weight and LC/MS peaks as computed by the spearman correlation coefficient.
