# Supplementary material for: Data Processing Thresholds for Abundance and Sparsity and Missed Biological Insights in an Untargeted Chemical Analysis of Blood Specimens for Exposomics
Source: Front Public Health. 2021 Jun 10;9:653599. doi: 10.3389/fpubh.2021.653599 (PMC8222544; doi:10.3389/fpubh.2021.653599)
Supplement: Supplementary Figure 1 — A negligible drift in the ESI signal across 499 files. Calibration compound (m/z = 922.0098) was injected on a constant flow rate by a secondary HPLC pump directly into the ESI ion-source. In case of a significant signal drift, a gray to black gradient in this plot would appear according to the injection order. Absence of such gradient shows a negligible signal drift in the study. [file Data_Sheet_1.pdf]

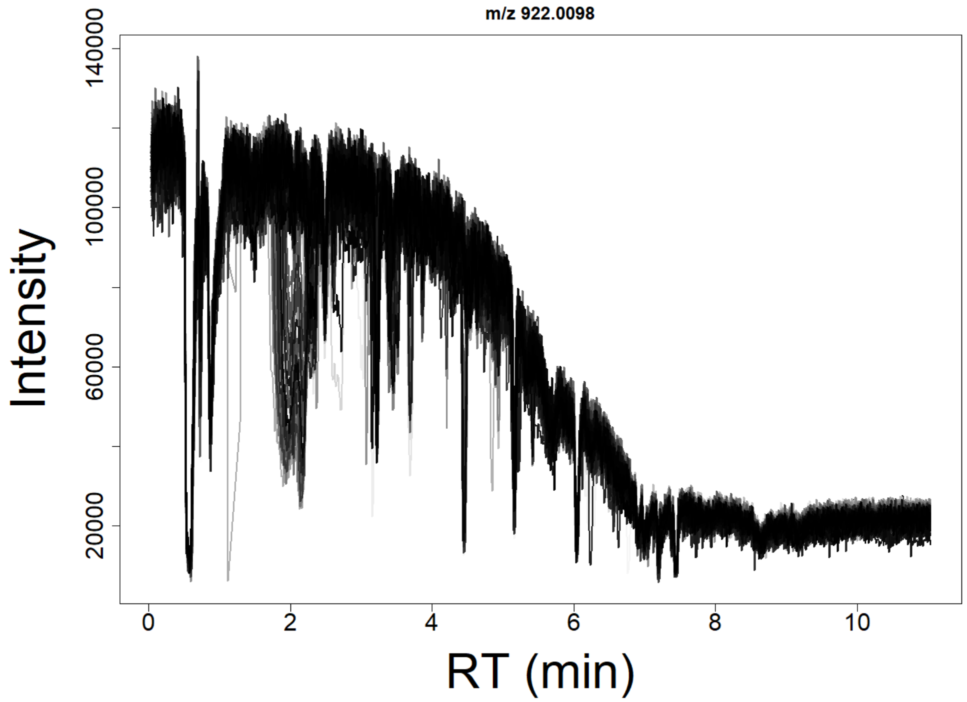

Figure S1 : A negligible drift in the ESI signal across 499 files. Calibration compound (m/z = 922.0098) was injected on a constant flow rate by a secondary HPLC pump directly into the ESI ion-source. In case of a significant signal drift, a grey to black gradient in this plot would appear according to the injection order. Absence of such gradient shows a negligible signal drift in the study.

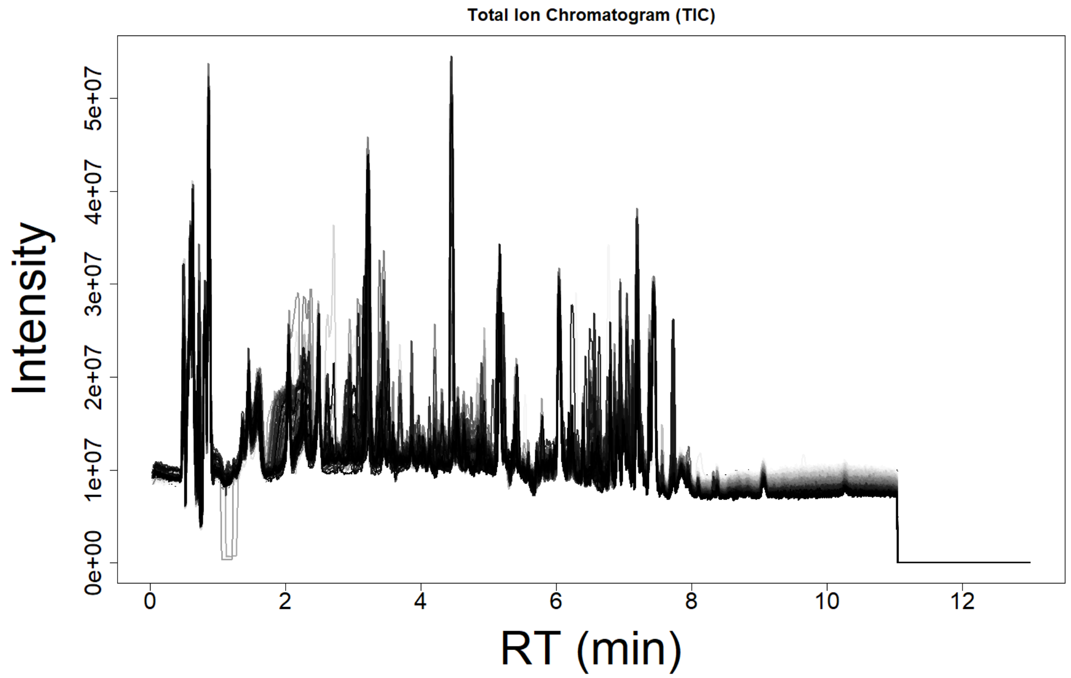

Figure S2 : A negligible drift in the ESI signal across 499 files as demonstrated by overlaying total ion chromatograms. . In case of a significant signal drift, a grey to black gradient in this plot would appear according to the injection order. Absence of such gradient shows a negligible signal drift in the study.

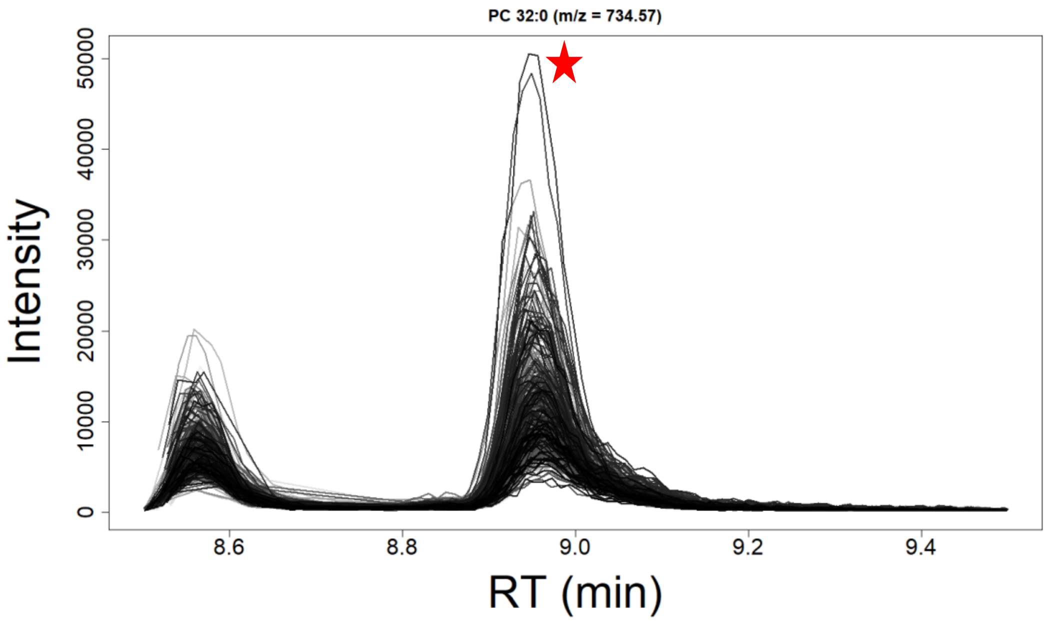

Figure S3 : Demonstration of a negligible drift in retention time for the peak of PC 32:0 (m/z 734.57).

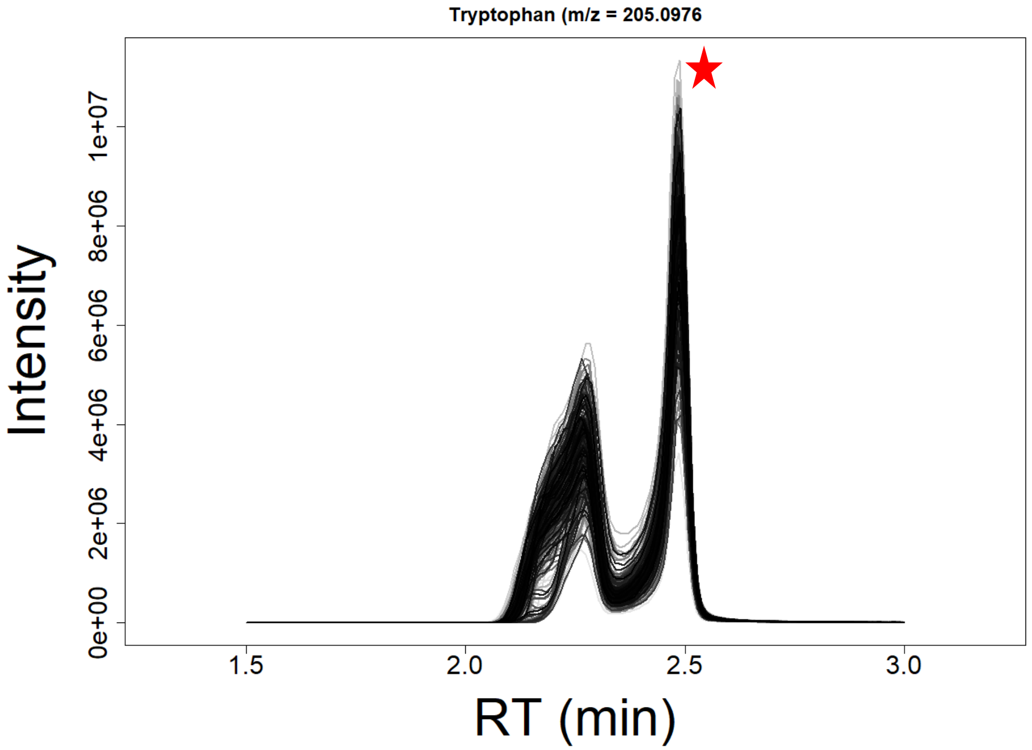

Figure S4 : Demonstration of a negligible drift in retention time for the peak of tryptophan (m/z 205.0976).

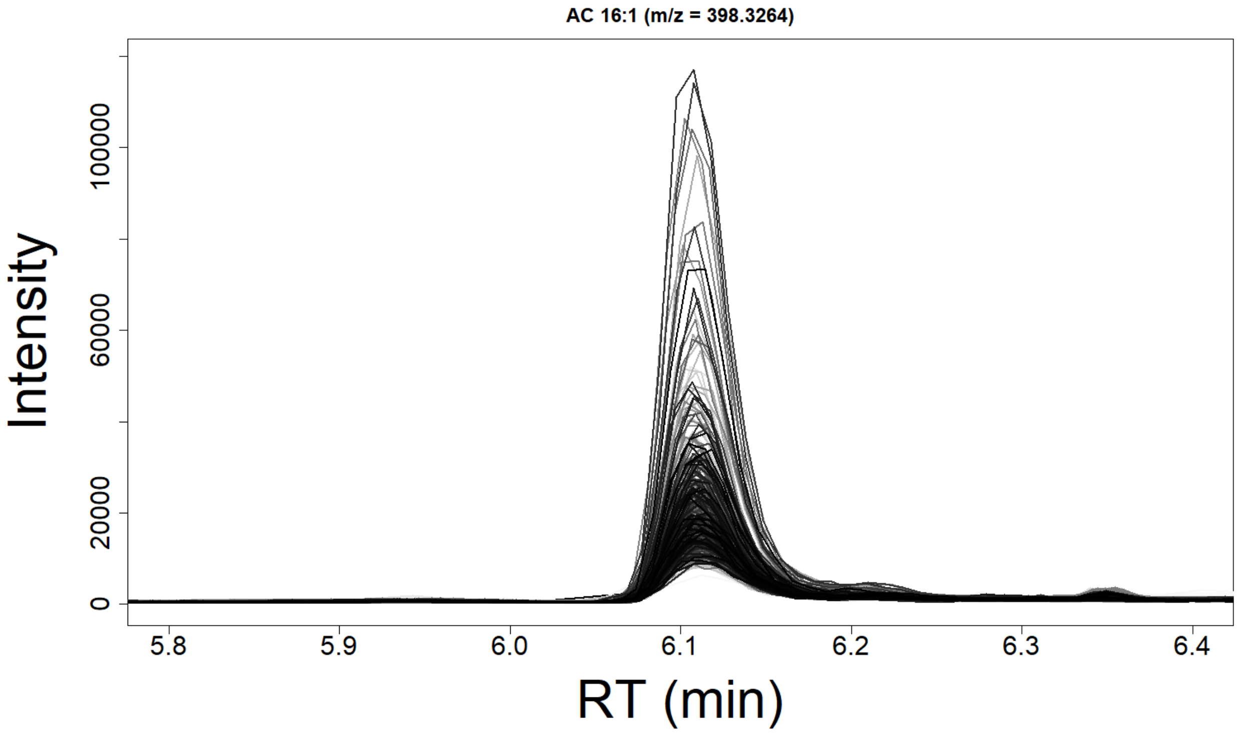

Figure S5: Demonstration of a negligible drift in retention time for the peak of AC 16:1 (m/z 398.3264).
